# Whole Organism Genome Editing: Targeted Large DNA Insertion via ObLiGaRe Nonhomologous End-Joining *in Vivo* Capture

**DOI:** 10.1534/g3.115.019901

**Published:** 2015-07-01

**Authors:** Yutaka Yamamoto, Jacob Bliss, Susan A. Gerbi

**Affiliations:** Brown University Division of Biology and Medicine, Department of Molecular Biology, Cell Biology and Biochemistry, Providence, Rhode Island 02912

**Keywords:** genome editing, nonhomologous end-joining (NHEJ), ObLiGaRe

## Abstract

Targeted gene insertion is a goal of genome editing and has been performed in cultured cells but only in a handful of whole organisms. The existing method to integrate foreign DNA using the homologous recombination pathway is inherently low efficiency, and many systems are refractory to this method. Several additional manipulations have been developed to gain greater efficiency by suppressing the competing dominant repair pathway of nonhomologous end-joining. However, this can be laborious and in practice limits the range of hosts where the method is applicable. Here, we use the preferred pathway of nonhomologous end-joining (used previously to create indels for gene inactivation) for precise integration of large DNA into the specified genomic target site of an intact animal. Our method uses site-specific cleavage, end-capture of cohesive ends, and obligate ligation-gated recombination. This approach is straight-forward and yields high efficiency without additional gene manipulations; therefore it is easily applicable to a much broader range of organisms. We demonstrate its application to the fungus fly *Sciara coprophila* where a transformation system has not existed before. We integrated a 6.5 kb transgene precisely at the desired genomic target site of *Sciara* using this method. This provides the foundation for future experiments to explore the unique genetic features of this organism. Similarly, the method described here will allow insertion of large pieces of DNA into a diverse group of organisms for studies of their genetic attributes.

The development of genome editing with targetable nucleases (Carroll 2014) has the potential to fully realize the power of genetic approaches in any organism, including nonmodel species that previously were refractory to study. Despite the rapid advances in this technology (Urnov *et al.* 2010; Gaj *et al.* 2013; Carroll 2014), the goal of efficient and precise insertion of large pieces of DNA into the target site has been difficult to achieve. Currently, targeted gene insertion relies on the homologous recombination (HR) pathway at a double-strand break (dsb) induced by various molecular scissors. Although this method can precisely integrate transgenes from 8 to 15 kb in cultured cells (Moehle *et al.* 2007; Jiang *et al.* 2013), the efficiency is not high enough for targeted gene insertion within genomes of whole organisms, especially where large-scale screening can be difficult. So far, targeted DNA insertion by HR has only been reported in a few whole organisms. We reasoned that the nonhomologous end-joining (NHEJ) repair pathway, which is preferentially used rather than HR by most organisms (Bozas *et al.* 2009), should be more efficient for targeted gene insertion in whole organisms. Typically the error-prone nature of NHEJ in the absence of donor DNA results in small indels that cause gene disruption at the target site. We report here the first example of precise insertion of large DNA into a targeted location in the genome of a whole animal via NHEJ.

## Materials and Methods

Male embryos of the fungus fly *Sciara coprophila* were injected with DNA, the adult males that emerged were crossed with *Sciara* adult females, and the G1 transgenic progeny were selected by their resistance to the antibiotic Blasticidin. The donor plasmid (2660iv pIDT-K) for somatic integration was constructed to contain the nt 2660 ZFN target sites, AscI, NdeI sites, Lox, attP, and FRT sites (the latter three for use in future applications such as cassette exchange) that were synthesized and cloned into the vector pIDTSmart-Kan (IDT). The 2660iv pIDT-K plasmid was further modified by insertion of 3XP3-TATA-TagYFP-PolyA and hr5-ie1-BlasR-PolyA as selectable markers to assay transgenic progeny after germline integration. Experimental samples were analyzed by polymerase chain reaction (PCR), genomic Southern blots, and sequencing.

### Data Availability

Strains and unique research materials are available upon request. Additional details for the *Materials and Methods* are available in the Supporting Information, File S1 along with Figure S1, Figure S2, and Figure S3.

## Results

As a starting point for site-specific insertion of DNA, molecular scissors are used to create a DNA fragment with overhanging cohesive ends. For our experiments we chose to use zinc finger nucleases (ZFNs) as the molecular scissors where target-site specificity is imparted by the zinc fingers and target cleavage is accomplished by Fok1 nuclease. Alternatively, TALENS or CRISPR coupled with Fok1 nuclease (Guilinger *et al.* 2014; Tsai *et al.* 2014) could be used as the molecular scissors. The short DNA fragment with compatible overhangs created by the double-strand break induced by ZFNs can be ligated *in vivo* (“end-capture”) through NHEJ but the efficiency is not greater than for HR (Orlando *et al.* 2010; Cristea *et al.* 2013; Weinthal *et al.* 2013). Consistent with these reports, end-capture mediated gene integration did not give us any greater efficiency compared with gene integration via HR in *Drosophila melanogaster* (Figure S1). Moreover, because we were not successful in integrating the marker gene via the ZFN-induced HR pathway in *Sciara* (Y. Yamamoto, unpublished results), it is unlikely that end-capture alone would give enough efficiency to successfully integrate the marker gene into the *Sciara* genome. Therefore, we investigated whether we could obtain high efficiency and fidelity for insertion of large DNA into an intact animal using obligate ligation-gated recombination (ObLiGaRe) that has been used recently for cultured cells (Maresca *et al.* 2013) for NHEJ. In this approach, the donor plasmid contains the same ZFN site as the target site to allow end-capture of the overhanging ends after digestion, but the ZFN binding sites in the donor plasmid are inverted relative to the host target sequence. We used this approach for site-specific insertion of large pieces of DNA into the genome of an intact organism (the lower dipteran fly *Sciara*). No transformation methods have existed previously to allow study of the many unique biological features of *Sciara*, which include site-specific DNA amplification in polytene chromosome DNA puffs, chromosome imprinting, monopolar spindle in male meiosis, X nondisjunction in male meiosis, embryonic chromosome elimination, germline limited chromosomes, and radiation resistance. In principle, the approach we describe can be used for any organism, suggesting the potential breadth of its application.

**Figure 1:**
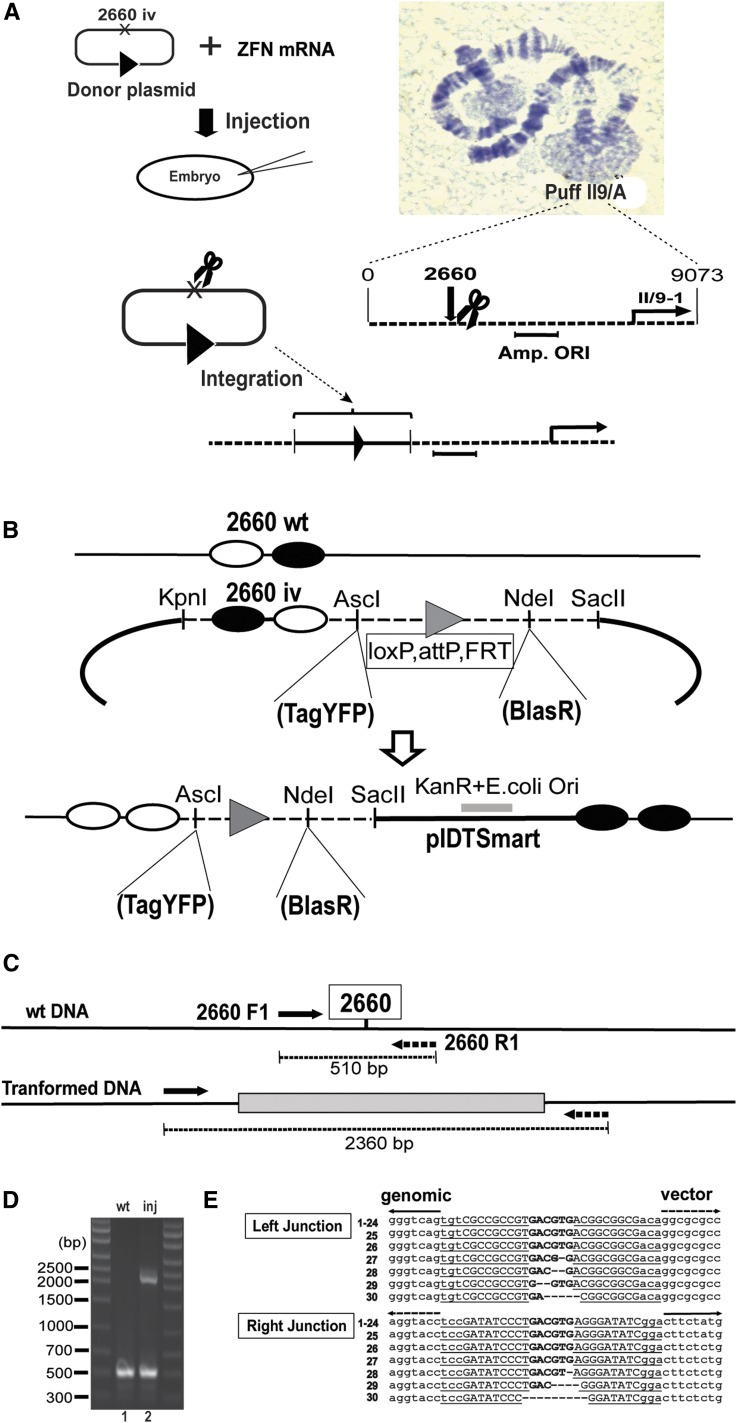
Somatic targeted gene insertion using ZFNs with ObLiGaRe. (A) Schematic for somatic integration at *Sciara* DNA puff II9/A, showing the nt 2660 cleavage site upstream of the amplification origin (Amp.ORI). (B) The principle of targeted gene insertion into nt 2660 of *Sciara* locus II/9A using an ObLiGaRe donor construct. ZFN binding sites (filled or open circles), wild-type (wt) or inverted sequence (iv) orientations, genomic DNA (solid line), cloning vector pIDTSmart (heavy black line) and linker sequence of the donor construct (dashed line) are indicated. The linker sequence (see File S1 for sequence) contains loxP, attP, and FRT site-specific recombination sites (gray triangle) for enhanced versatility and potential use in future experiments. (C) Polymerase chain reaction (PCR) primer pairs are shown along with the expected PCR products of 510 bp (wt) and 2360 bp (transformed) using genomic DNA from injected embryos. (D) Gel of the genomic PCR products (lane 1: wt, wild-type; lane 2: inj, injected). (E) Sequence alignment of the junction between the genomic DNA and the integrated donor DNA in clones containing the 2360 bp PCR fragment. The 12 bp inverted sequences of 2660iv are underlined and capital letters show the 9 bp ZFN-binding sites; the linker sequence at the ZFN target site is shown in bold. Each of the 30 clones that were sequenced was named with a number (indicated).

First, we examined the efficiency and fidelity for targeted integration in somatic cells of *Sciara*. We constructed a donor plasmid to target nt 2660 just upstream of the DNA amplification origin at *Sciara* DNA puff II/9A (Figure 1A) to allow future studies on *cis*-regulatory elements for locus-specific re-replication. The ZFN cleaves both the wild-type genomic target site and the inverted target site. When vector ends are captured at the target site, the same ZFNs cannot cleave the newly formed junction since formation of the homodimer is prevented (Doyon *et al.* 2011); as a result, DNA molecules accumulate with the transgene (Figure 1B and Figure S2). We coinjected the donor plasmid and ZFN mRNAs into embryos, extracted genomic DNA 3 days later, and carried out PCR (Figure 1C). In addition to the 510 bp PCR product from the wild-type DNA, a 2360 bp PCR product also was present, indicating successful integration of the donor construct at nt 2660 (Figure 1D). The 2360 bp fragment was gel purified, subcloned, and 30 independent randomly chosen clones were sequenced. Among the 30 clones, three carried small deletions in both right and left junctions and one clone had a small deletion at one junction. However, 26 clones were perfect with no junction alterations (Figure 1E). In addition to the high percentage of clones reflecting precise integration, we were impressed by the high amount (∼27%) of 2360 bp PCR product indicative of DNA integration without any selection compared to the 510 bp wild-type PCR fragment.

**Figure 2:**
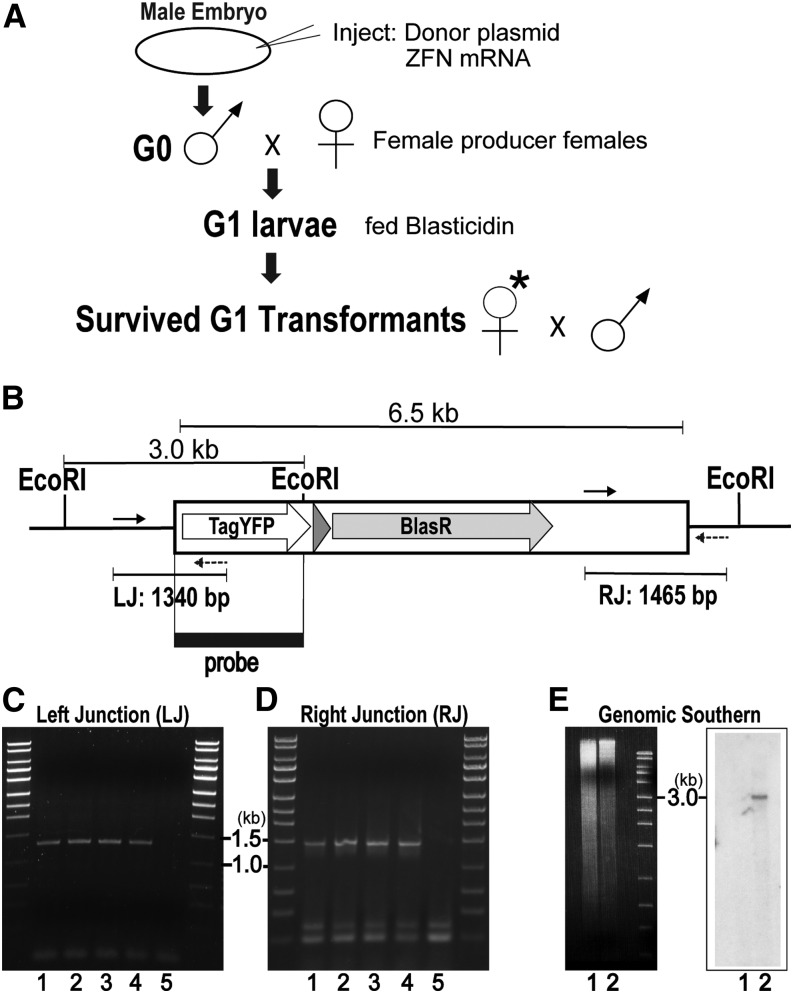
Germline inherited targeted gene insertion using ZFNs with ObLiGaRe. (A) Schematic of the injection and subsequent screening of the germline transgenes using Blasticidin resistance as the selection marker. The asterisk indicates a candidate transgenic G1 female. (B) The transgene for the germline integration contains two marker genes (3XP3-TATA-TagYFP-PolyA, hr5-ie1-BlasR-PolyA) and site-specific recombination target sequences loxP-attP-FRT (gray triangle) for potential use in future experiments. Polymerase chain reaction (PCR) fragments including junction between genomic DNA and inserted donor DNA are shown as a line with PCR primer-pairs indicated for the left junction (LJ: 1340 bp) and right junction (RJ: 1465 bp). The 1.5 kb DNA hybridization probe (black rectangle) is complementary to sequences within a 3.0 kb *Eco*R1 genomic fragment from transformed *Sciara*. (C, D) Gel photos of the genomic PCR products (Lanes 1-4: genomic DNA extracted from four independent transgenic lines; Lane 5: genomic DNA from uninjected control). (C) Left junction 1340 bp PCR products. (D) Right junction 1465 bp PCR products. (E) Left panel: Gel of *Eco*RI-digested genomic DNA before transfer. Right panel: Genomic Southern blot using the 1.5 kb fragment of the integration marker as the probe. Lane 1: uninjected control DNA, lane 2: mixture of genomic DNA from four independent transgenic lines.

On the basis of the promising results from somatic integration, we proceeded with germline integration. For this, we added two integration markers in the donor construct (3XP3-TATA-TagYFP-PolyA; hr5-ie1-BlasR-PolyA) (Figure 1B), which was coinjected into *Sciara* male embryos with the same ZFN mRNAs used above. In this experiment, the total DNA for integration was 6.5 kb (Figure 2B). The adult G0 male flies were crossed with wild-type females, and among 116 successful G0 crosses, we established eight independent transgenic lines (from eight individual G0 males) of Blasticidin resistant larvae (Figure 2A). Genomic DNA from four of these individual lines was characterized further. PCR spanning the nt 2660 target site gave the expected products of 1340 bp (Figure 2C, left junction) and 1465 bp (Figure 2D, right junction) for all four lines of independent transformants. Sequence analysis of the subcloned junction PCR fragments confirmed that the donor DNA fragment was integrated at nt 2660, and there were no alterations in the junction between the inserted DNA and genomic sequences. Thus, the four fly lines had high efficiency and perfect integration of the 6.5 kb DNA at the nt 2660 target site with no sequence changes. Furthermore, genomic Southern blot analysis of *Eco*RI-digested DNA from the four individual lines (combined) using an insert specific probe revealed only a single band of 3.0 kb (Figure 2E), confirming that there is no off-site integration. The integration of the transgene of all four lines appeared to be mono-allelic (Figure S3).

## Discussion

Targeted gene manipulations have become extremely popular using the latest development of molecular scissors. HR-mediated transgenesis is a powerful tool for gene manipulation such as DNA insertion into the genome. However, HR is the minor repair pathway and is less preferred in the cell than NHEJ that is the major repair pathway. Therefore, to enhance the use by the cell of the HR pathway, additional genetic manipulations often are employed such as use of the DNA ligase IV mutant or RNAi to suppress the NHEJ major repair pathway (Bozas *et al.* 2009, Maruyama *et al.* 2015, Chu *et al.* 2015). Nonetheless, these additional genetic manipulations give added complexity to the experiment, require more time, and these approaches are not always available in a variety of organisms. As a result, many organisms are refractory to DNA insertion in genome editing using HR. Although the preferred DNA repair pathway of NHEJ has been used widely in many organisms for targeted mutagenesis, generally its products are small insertions and small deletions. The integration of large DNA fragments via NHEJ has been inefficient as well as imprecise (Orlando *et al.* 2010; Cristea *et al.* 2013; Weinthal *et al.* 2013) and not widely used.

However, the use of NHEJ for targeted insertion is critical in systems refractory to HR. We show here that ObLiGaRe with NHEJ can be used to integrate large DNA (6.5 kb) into the desired genomic target site of whole animals by simply injecting the mixture of ZFN mRNAs and a donor plasmid into the embryo. In addition, DNA integration into the targeted chromosome can be screened by phenotypic markers that are integrated into the target site. This supercedes the necessity of screening with balancer chromosomes, mutations or deletions that are usually not available in most organisms. With ObLiGaRe, the integration efficiency into the germline genome was high enough (7%) for us to recover eight antibiotic resistant individual lines (from different G0 males) out of 116 successful G0 crosses. Moreover, the four lines we characterized all had precisely ligated junctions. Our method makes targeted mutagenesis possible in experimental systems like *Sciara* where genetic resources have been limited. In addition, the ability to integrate relatively long DNA fragments into a specified genomic target site with high efficiency combined with the ease of making the targeting donor will be welcomed even for organisms where targeting gene insertion through HR is routinely being used.

Because our previous attempts to obtain transgenic flies using HR with the same set of ZFN and marker genes as used here was unsuccessful (Y. Yamamoto, unpublished results), it is likely that the much greater efficiency derived from ObLiGaRe is the main factor for successful recovery of transgenic flies in *Sciara*. It has been reported that in cultured cells, the transformation efficiency with ObLiGaRe is five times greater than using HR (Maresca *et al.* 2013). Our paper is the first report of using ObLiGaRe in a whole organism. ObLiGaRe utilizes the fact that custom designed nucleases (ZFNs or TALENs) use the FokI endonuclease domain, which has to form a dimer to be active. Two separate DNA binding sites are required to make the inverted target site for the ObLiGaRe format (Maresca *et al.* 2013). Therefore, CRISPER/Cas9 in its original form is not compatible with ObLiGaRe. However, the newly developed dimeric CRISPR-FokI nuclease could be very useful as molecular scissors (Guilinger *et al.* 2014; Tsai *et al.* 2014), providing an alternative to ZFNs or TALENs for future applications of ObLiGaRe.

## Supplementary Material

Supporting Information
