# Supplementary material for: Whole Organism Genome Editing: Targeted Large DNA Insertion via ObLiGaRe Nonhomologous End-Joining in Vivo Capture
Source: G3 (Bethesda). 2015 Jul 1;5(9):1843–7. doi: 10.1534/g3.115.019901 (PMC4555220; doi:10.1534/g3.115.019901)
Supplement: Supporting Information [file supp_5_9_1843__index.html]

Whole Organism Genome Editing: Targeted Large DNA Insertion via ObLiGaRe Nonhomologous End-Joining in Vivo Capture — Supporting Information 

# Whole Organism Genome Editing: Targeted Large DNA Insertion via ObLiGaRe Nonhomologous End-Joining *in Vivo* Capture

## Supporting Information for Yamamoto, Bliss, and Gerbi, 2015

**Files in this Data Supplement:**

- Supporting Information - Figures S1-S3 and File S1 (PDF, 912 KB)
- Figure S1 - Comparison of transgenic DNA insertion in *Drosophila* via homologous recombination compared to nonhomologous end-joining. (PDF, 675 KB)
- Figure S2 - ObLiGaRe construction. (PDF, 171 KB)
- Figure S3 - Mono-allelic integration of the 6.5 kb transgene in the *Sciara* genome. (PDF, 199 KB)
- File S1 - Supplemental Materials and Methods (PDF, 105 KB)
