# Supplementary material for: Whole Organism Genome Editing: Targeted Large DNA Insertion via ObLiGaRe Nonhomologous End-Joining in Vivo Capture
Source: G3 (Bethesda). 2015 Jul 1;5(9):1843–7. doi: 10.1534/g3.115.019901 (PMC4555220; doi:10.1534/g3.115.019901)
Supplement: Supporting Information [file supp_g3.115.019901_FigureS2.pdf]

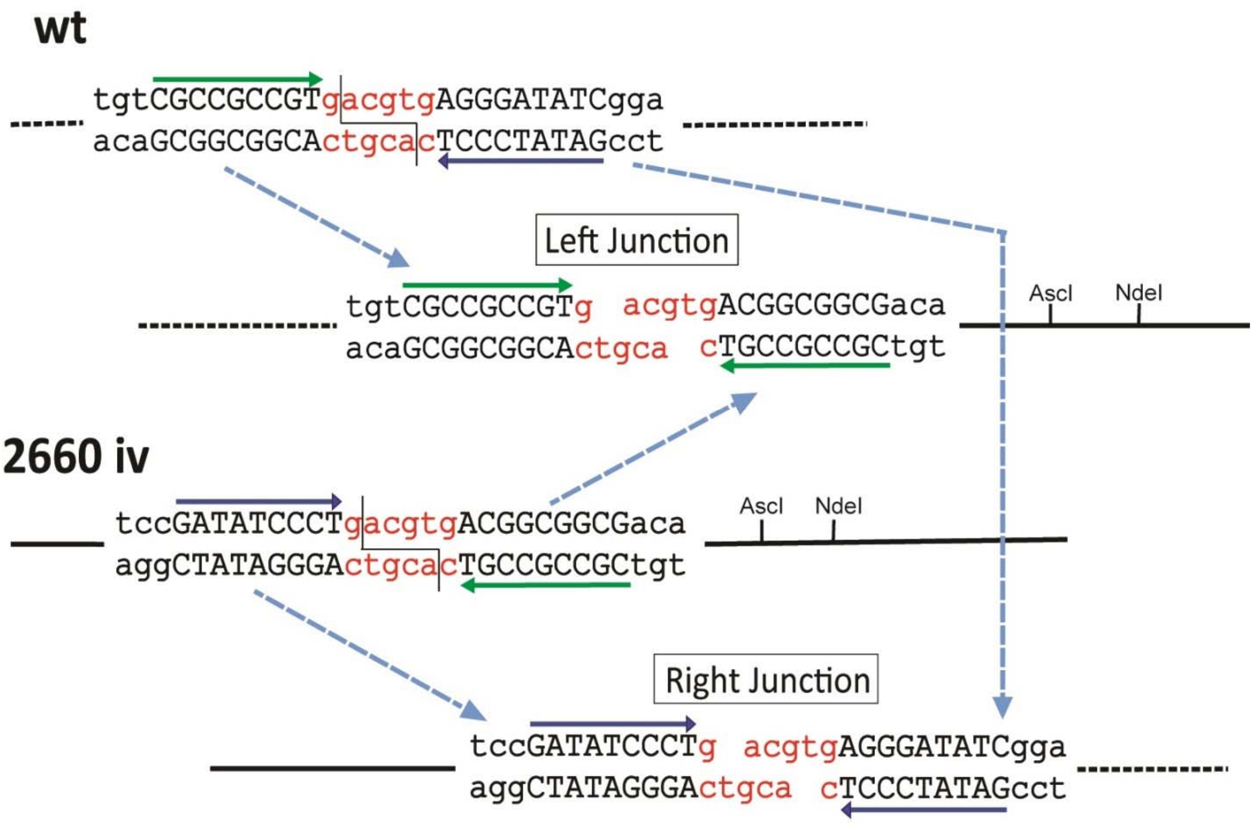

Figure S2 ObLiGaRe construction. Genomic DNA is shown as dotted lines and vector DNA is shown as solid lines. Two unique restriction sites (AscI and NdeI) are shown at the right side of the vector as orientation markers. Two blocks of 12 bp sequences including the 9 bp ZFN target sites are inverted to make 2660iv. The linker sequence is shown in red; it is between the two flanking ZFN binding sequences that are shown in black capital letters. Compatible overhangs at the ends created by the same ZFN pairs from the genomic target and the inverted donor target are ligated *in vivo* by ObLiGaRe.
