# Supplementary material for: Whole Organism Genome Editing: Targeted Large DNA Insertion via ObLiGaRe Nonhomologous End-Joining in Vivo Capture
Source: G3 (Bethesda). 2015 Jul 1;5(9):1843–7. doi: 10.1534/g3.115.019901 (PMC4555220; doi:10.1534/g3.115.019901)
Supplement: Supporting Information [file supp_g3.115.019901_FigureS1.pdf]

Supplemental Figures

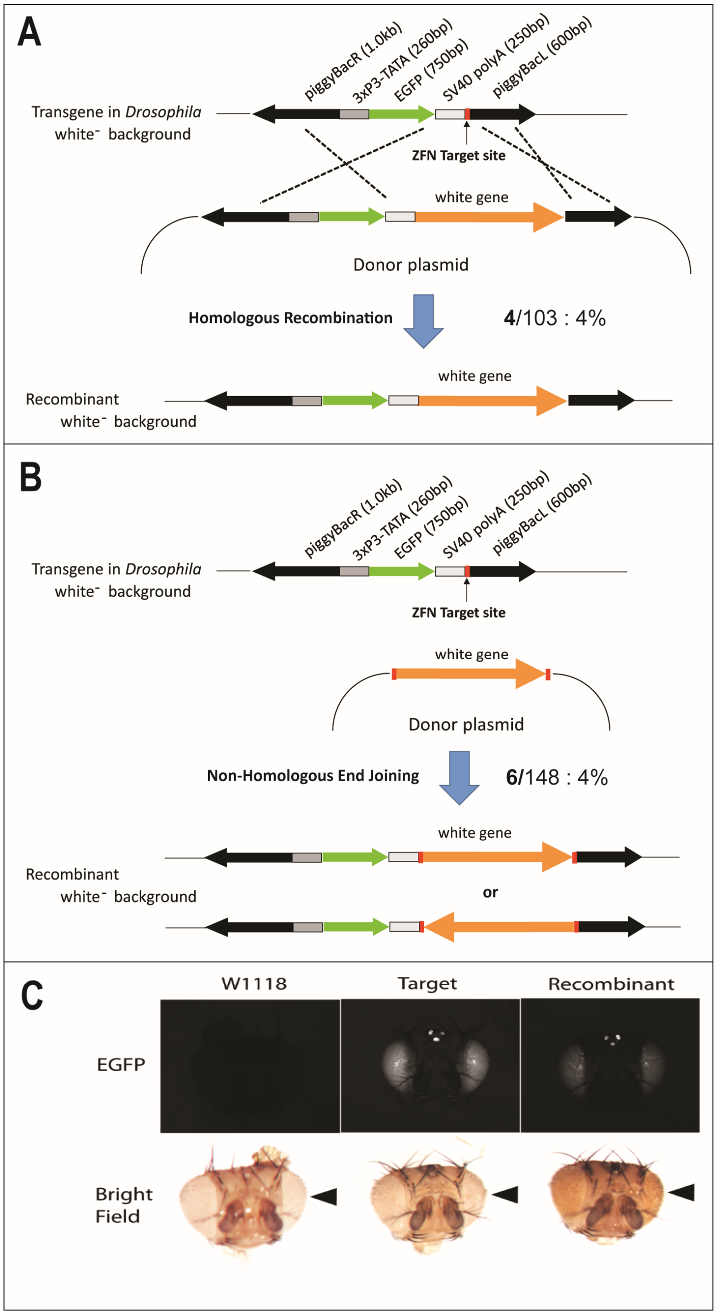

Figure S1 Comparison of transgenic DNA insertion in *Drosophila* via homologous recombination compared to nonhomologous end-joining. An artificial ZFN target site (red rectangle) was introduced by transformation with a standard piggyBac vector carrying an artificial ZFN target site (piggyBac[3XP3-EGFPafm],ZFN-T) that inserted on the third chromosome of *Drosophila melanogaster*. The mini-white gene was integrated into the ZFN target site either via

homologous recombination or via nonhomologous end-joining, by co-injecting a pair of ZFN expression vector and a donor plasmid into the eggs.

(A) The donor construct (piggyBac[3XP3-EGFPafm], mini-white) was designed to integrate the mini-white gene (orange arrow) by homologous recombination (HR) using the flanking piggyBac vector sequences (black arrows) that are present in both the donor plasmid (middle line) and the host genome (top line). The measured frequency of mini-white gene insertion by HR was 4%. The product of HR is shown on the bottom line where the mini-white gene has replaced the genomic zinc finger nuclease (ZFN) target sequence upon integration.

(B) The donor plasmid (pBS-miniwhite; middle line) was designed to introduce the mini-white gene (orange arrow) into the host genome (top line) by nonhomologous end-joining (NHEJ). ZFNs cleave two ZFN target sites (red rectangles) in the donor plasmid and genomic target site. Subsequently, the mini-white gene with compatible cohesive ends was ligated *in vivo* into the genomic target site that was cleaved by the same ZFNs ("end-capture"). After end-capture of the mini-white gene, the ZFN target sequences flanking this marker gene are restored and can be re-cleaved by the same ZFN. The products of this NHEJ are shown on the bottom line, and the measured frequency of mini-white gene insertion by NHEJ was 4%.

(C) *Drosophila* carrying the ZFN target site were identified by the EGFP signal in the adult eye. The host flies had white eyes (bright field). Recombinants were identified by the orange eye color. The EGFP signal of the recombinant flies was partially masked due to the eye pigment from mini-white gene. Via HR we recovered 4 individual orange eye colored flies out of 103 successful G0 crosses. Via NHEJ, we recovered 6 individual orange eyed G1 flies from 148 fertile G0 crosses.
