# Supplementary material for: Whole Organism Genome Editing: Targeted Large DNA Insertion via ObLiGaRe Nonhomologous End-Joining in Vivo Capture
Source: G3 (Bethesda). 2015 Jul 1;5(9):1843–7. doi: 10.1534/g3.115.019901 (PMC4555220; doi:10.1534/g3.115.019901)
Supplement: Supporting Information [file supp_g3.115.019901_FigureS3.pdf]

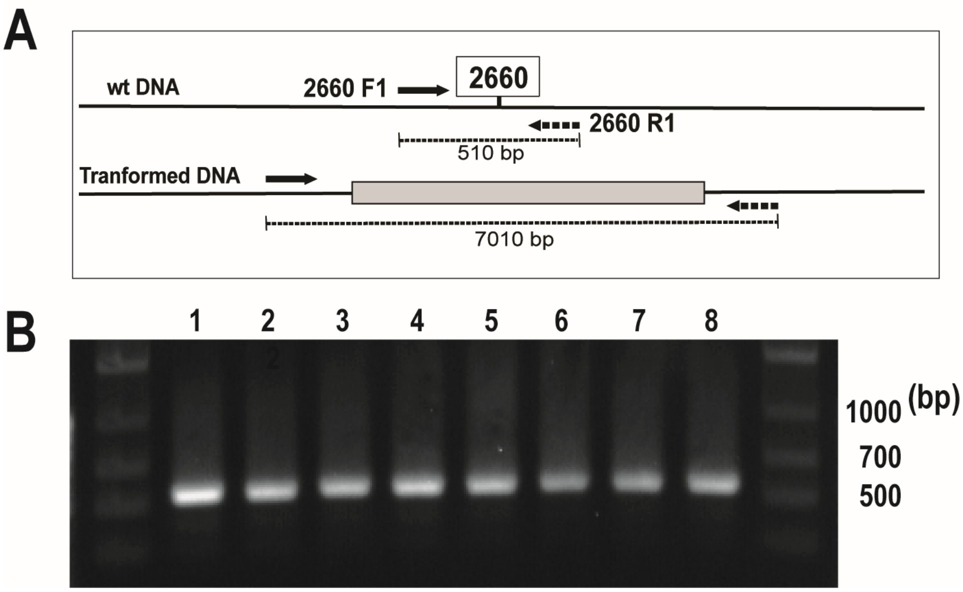

Figure S3 Mono-allelic integration of the 6.5 kb transgene in the *Sciara* genome. To determine whether the germline integration of the transgene was mono-allelic or bi-allelic, genomic PCR with DNAs isolated from individual G1 transformed female flies was performed. As shown in Figure 2A, male *Sciara* embryos were injected and upon reaching adulthood, these G0 males were crossed with wild type female-producing female flies. The G1 larvae (all female) from this cross were screened for Blasticidin resistance, and were mated to create eight independent transformant lines. After the G1 female transformants flies were crossed with wild type males and had laid eggs, genomic DNA was isolated from the G1 females and used as the template for PCR. **(A)** The PCR products using primer set 2660 F1 and 2660 R1 are 510 bp (from a wild type chromosome) and 7010 bp (from a chromosome after DNA insertion). **(B)** Gel electrophoresis of the PCR products revealed that all 8 independent transformant lines had the 510 bp product derived from a wild type chromosome (no insert), indicating that all the transgenic lines were mono-allelic (one wild type allele as shown and one allele with the transgenic DNA insertion).
