# Supplementary material for: Whole Organism Genome Editing: Targeted Large DNA Insertion via ObLiGaRe Nonhomologous End-Joining in Vivo Capture
Source: G3 (Bethesda). 2015 Jul 1;5(9):1843–7. doi: 10.1534/g3.115.019901 (PMC4555220; doi:10.1534/g3.115.019901)
Supplement: Supporting Information [file supp_g3.115.019901_FileS1.pdf]

File S1

SUPPLEMENTAL MATERIALS AND METHODS

Injection and antibiotic selection

Eggs from male producer females of the fungus fly *Sciara coprophila* were collected on an agar plate at 18^0C and injected within two hours. Donor DNA for the injection was prepared using the PureLink HiPure Plasmid Midi Purification kit (Invitrogen), and ZFN mRNAs were synthesized using a mMESSAGE mMACHINE Kit (Ambion) and purified by phenol/chloroform extraction. The concentration of the donor DNA and ZFN mRNA were adjusted to 1 µg/µl and 200 ng/µl final in water.

For the germline transformation experiment, the injection was carried out aiming to obtain fifty fertile adult survivors per injection. Injected eggs (G0) were raised at 18^0C and emerged adult males were crossed with a total of six female producer females for three days. All G1 eggs recovered from the G0 cross were incubated at 18^0C in a humid chamber for five days and transferred onto selection agar plates (12 g/500 ml) containing 10 µg/ml of Blasticidin-S hydrochloride (A.G. Scientific, Inc.). Emerged G1 larvae were kept on the same selection plate until control larvae (uninjected) died completely. The surviving G1 larvae were transferred to non-selective agar plates and fed until they hatched. The hatched G1 flies were mated; after they produced eggs, they were frozen to be processed for genomic DNA extraction. We established eight independent transgenic lines (from eight individual G0 males) of Blasticidin resistant larvae. The amount of labor precluded amplifying all eight lines, so we have characterized the molecular details of the transgene in the first four lines that were established as stocks.

Estimation of molecules carrying the transgene after somatic integration

For the somatic integration experiment, ~300 embryos were injected and incubated at 18^0C. Two days after the injection, properly developing embryos were collected and pooled to store at -20^0C. Genomic DNA was extracted from 100 pooled embryos and used for PCR (Figure 1). A serial dilution was made from the genomic PCR reaction and intensity of the bands in the gel was compared with the intensity of the size marker (1 kb ladder, Promega). The concentration of the 2.4 kb and 510 bp bands determined by this method were 160 ng/ul and 450 ng/ul respectively, representing a ratio of **1** (2.4kb): **13** (510bp) for the number of molecule in those two bands. The genomic DNA was extracted from entire embryo after injection, however, it is estimated that the injected material will spread only ~1/4 to ~1/3 of the entire length of the egg (materials are injected at the posterior end of the egg, the same as for the germline transgenesis). Therefore, only ~1/4 to ~1/3 of genomic DNA was expected to be exposed to the injected

DNA. Taken this into account, we estimate that ~27% of the genomic DNA exposed to the injected DNA carry the transgene after somatic integration.

Zinc Finger Nucleases

ZFNs with obligated heterodimer nuclease to target nucleotide position 2660 of amplification locus II/9A were kindly provided by Sangamo BioSciences and were engineered and validated as described (Urnov et al. 2010). The ZFN binding sequence for target site 2660 at locus II/9A of *Sciara* had 9 bp at the left half site and 9 bp at the right half site separated by a 6 bp linker (bold):

CGCCGCCGT-**GACGTG-**AGGGATATC

The targeting donor construct (2660iv pIDT-K) for somatic integration

A linker of 393 bp containing the inverted 2660 ZFN target sites, AscI, NdeI sites, Lox, attP and FRT sites (the latter three for potential use in future experiments) was synthesized and cloned into pIDTSmart-Kan (IDT). This 393 bp fragment had the sequence:

[1]GGTACCTCCGATATCCTGACGTGACGGCGGCGACAGGCGCGCCTGTCGCCGCCGTGGCGCGCCATAACTTCG
TATAATGTATGCTATACGAAGTTATAAATAATGATTTTATTTTGACTGATAGTGACCTGTTCGTTGCAACACATTGA
TGAGCAATGCTTTTTTATAATGCCAACTTTGTACAAAAAAGCTGAACGAGAAACGTAAAATGATATAAATATCAATA
TATTAAATTAGATTTTGCATAAAAAACAGACTACATAATACTGTAAAACACAACATATCCAGTCACTATGAATCAAC
TACTTAGATGGTATTAGTGACCTGTAGAAGTTCCTATTCCGAAGTTCCTATTCTCTAGAAAGTATAGGAACTTCCA
TATGATATCCGCGG[393]

The targeting donor construct (2660iv pIDT-K, TagYFP+BlasR) for germline transgenesis

For germline transgenesis, the two selectable marker genes 3XP3-TATA-TagYFP-PolyA and hr5-ie1-BlasR-PolyA were cloned into the AscI site and NdeI site of 2660iv pIDT-K, respectively. See Figure 1 for a map of this donor construct.

<u>Construction of 3XP3-TATA-TagYFP-PolyA</u>:

The 750 bp TagYFP coding sequence was amplified from pTagYFP-C (Evrogen) using primer pairs P3-TagYFP F1 (GCCCGGGATCCACCGGTCGCCACCATGGTTAGCAAAGGCGAGGAGCTGTTCGCCGGC) and TagYFP 3R1 (AGAGTCGCGGCCGCTTTACCGGTACAGCTCGTCCATGCCGTGGGTGTGGC.

A 1 kb fragment containing the 3XP3-TATA promoter was amplified from pBac[3XP3-TATA-EGFP-PolyA afm] (kindly provided from Dr. Alfred M. Handler, USDA - Gainesville) using primer pairs PstI F1

(CCTACTGCAGGTCATCACAGAACACATTTGGTCTAGCGTGTCCACTCCGCC) and

P3-TagYFP R1

(GGCGGAGTGGACACGCTAGACCAAATGTGTTCTGTGATGACCTGCAGTAGG). The 1 kb promoter-containing fragment and the 750 bp tagYFP fragment were mixed together and amplified with primer sets PstI F1 (see above for sequence) and TagYFP 3R1 (GCGCCTGTAGCCACACCCACGGCATGGACGAGCTGTACCGGTAAAGCGGCCGCAAGAA). The 1.8 kb product was purified, digested with PstI and NotI, and cloned into the PstI-Not I site of pBac[3XP3-TATA-EGFP-PolyA afm]. This cloning product was called pBac[3XP3-TATA-TagYFP-PolyA afm], which had TagYFP rather than the starting EGFP, and 3XP3-TATA-TagYFP Poly A from this clone was amplified using

primer-set AscI-TagYFP F1

(ATATATGGCGCGCCGATGTTCCCACTGGCCTGGAGCGACTGTTTTTCAGTACTTCCGGTATCTCGCG) and AscI-TagYFP-R1 (TTAATAGGCGCGCCGTACGCGTATCGATAAGCTTTAAGATACATTGATGAG) for cloning into the AscI site of 2660iv pIDT-K.

<u>Construction of hr5-ie1-BlasR-PolyA:</u>

The Blasticidin coding sequence was amplified with BlasS SacII F1 (AATTACCGCGGATAAAATGGCCAAGCCTTTGTCTCAAGAAGAATCCACCCTC ATT) and BlasS BamHI R3 (AATTGGATCCGCCCTCCCACACATAACCAGAGGGCAGCAATTCACGA)

from pcDNA6/V5-His-A (Invitrogen) and then cloned into the SacII-BamHI site of pIE1-3 carrying piggyBac transposase (kindly provided by Dr. Craig J. Coates, Texas A&M). The Hr5-ie1-Blasticidin coding sequence-polyA sequence of this clone was amplified with primer set pIE AscI F1 and pIE AscI-R1 and cloned into the AscI site of plasmid 2660iv pIDT-K for germline transgenesis. The sequences for these primer-sets are:

pIE Ascl F1:

AATTGGCGCGCCCGCGTAAAACACAATCAAGTATGAGTCATAAGCTGAT, and

plE Fse Rl:

ATATGGCCGGCCAAGCTTAAAAGTAGGAGGAACGGGCATACTCTT

Genomic PCR

All PCR was performed with Q5 High-Fidelity DNA Polymerase (New England BioLabs), based on the manufacturer's recommended conditions where annealing and extension are done simultaneously in the two-step method. PCR for the somatic integration used 103^{0}C (1 min), 40 cycles of 103^{0}C (10 sec) and 72^{0}C (3 min), and

concluded with 72^{0}C (6 min) followed by 4^{0}C. PCR for the germline integration used 103^{0}C (1 min), 40 cycles of 103^{0}C (10 sec), 55^{0}C (10 sec) and 72^{0}C (45 sec), and concluded with 72^{0}C (2 min) followed by 4^{0}C.

Primers:

2660 F1 (GAACACAATGGAGTCGAAGCATAGCAAGATGGGGTGCG)

2660 R1 (GAACGAGCCGAAGGCGAGTGGAGTAATAACACAAGCC)

pSMART F1 (CGGCGATCGCGTATTTCGTCTCGC)

pSMART R1 (GTACAGCTCGTCCATGCCGTGGGTG)

Genomic Southern blots and sequencing

10 µg of EcoRI digested genomic DNA was separated in a 1% agarose gel in 1XTBE, then transferred onto Hybond-N membrane (Amersham) with the alkaline method (0.4 N NaOH). The 1.5 kb KpnI-EcoRI fragment was isolated from (2660iv pIDT-K, TagYFP+BlasR) and labeled with the RadPrime DNA Labeling System (Invitrogen). Hybridization was carried out in Church buffer at 65^{0}C overnight and washed in 2x SSC, 0.1% SDS, 15 min x2 at 65^{0}C, and 0.1X SSC, 0.1% SDS, 15 min x2 at 65^{0}C. The gel purified fragments after somatic integration (2360 bp) or germline integration (left junction: 1340 bp, right junction: 1465 bp) were subcloned into the pCR Blunt II-TOPO vector (Invitrogen) and sequenced. The somatic 510 bp fragment was also cloned and sequenced as a control.

Comparison of DNA insertion by HR and NHEJ in *Drosophila*

Drosophila melanogaster was transformed with a piggyBac vector carrying an artificial ZFN target site (piggyBac[3XP3-EGFPafm],ZFN-T) that was constructed as follows.

A linker containing an artificial ZFN target site was synthesized (IDT) with the sequence: AATTGGCCGGCCCGCTACCCCGACCATGAAGCAGCAGAATTCGGCGCGCCGGCCGGCCATAT

where the underlined sequence is the 24 bp ZFN target site (Urnov et al, 2005). This linker was cloned into FseI site of piggyBac[3XP3-EGFPafm] (gift from Dr. A.M. Handler) (Horn and Wimmer, 2000). *Drosophila melanogaster* (white minus) was transformed with this construct (piggyBac[3XP3-EGFPafm],ZFN-T), and a transformant where integration occurred on the third chromosome served as the host for the subsequent experiments with HR and NHEJ.

The donor construct (piggyBac[3XP3-EGFPafm], mini-white) was designed to integrate the mini-white gene (orange arrow) by homologous recombination (HR) using the flanking piggyBac vector sequences (black arrows) that are present in both the donor plasmid (middle line) and the host genome (top line)

The donors piggyBac[3XP3-EGFPafm], mini-white and pBS-miniwhite both contained the mini-white gene for integration into the *Drosophila* genome by HR or NHEJ, respectively. They were constructed as follows. The first step for construction of both donors was the preparation of pSL11180-miniwhite. It had the 4.6 kb mini-white HindIII-EcoRI fragment that was isolated from pP{CaSpeR-4} (FlyBase: http://flybase.org/reports/FBtp0000163.html) and was cloned into the HindII-EcoRI sites of pSL11180af (gift from Dr. A.M. Handler). Subsequently, for the HR donor, the mini-white gene was excised by FseI and cloned into the FseI site of piggyBac[3XP3-EGFPafm] to create piggyBac[3XP3-EGFPafm], mini-white. Instead, for construction of pBS-miniwhite for use in NHEJ integration, pBS-AscI was created to insert the mini-white gene at the AscI site between two ZFN-Ts. For this purpose, a linker (KpnI-ZFN-T-AscI-ZFN-T-SacI) was synthesized (IDT); it contained an AscI site and two artificial ZFN target sites and had the sequence:

GGTACCCGCTACCCCGACCATGAAGCAGCAGGCGCGCCCGCTACCCCGACCATGAAGCAGCAGAGCTC

This linker was cloned into the KpnI and SacI sites of pBluescript II SK (Stratagene) to create pBS-AscI. Next, the mini-white gene was excised from pSL11180-miniwhite using AscI and cloned into the AscI site of pBS-AscI to create pBS-miniwhite.

The ZFNs expression plasmid (gift from Dr. F.D. Urnov) was coinjected with the donor constructs for the HR and NHEJ experiments in *Drosophila* (Figure S1). The coding sequences of the entire ZFN-R and ZFN-L were amplified from the original clones in pVAX (Invitrogen) using primer sets ZFN SacII and ZFN AscI and cloned into the SacII-AscI sites of expression vector pIEX3 (gift from Dr. C.J. Coates) (Mohammed and Coates, 2004). The ZFN SacII and ZFN AscI primer sets had the sequences:

ZFN SacII: ATGACCGCGGATAAAATGGCCCCCAAGAAGAAGAGGAAG

ZFN AscI: ACGTGGCGCGCCTTAAAAGTTTATCTCGCCGTTATTAAATT

The mini-white gene was integrated into the ZFN target site either via HR or NHEJ. For each injection, 1 μg/μl of donor construct DNA and 200 ng/ml of ZFN expression constructs were used.

SUPPLEMENTAL REFERENCES

Horn, C., Wimmer, E.A., 2000. A versatile vector set for animal transgenesis. *Dev. Genes Evol.* **210**: 630-637.

Mohammed, A., Coates, C.J., 2004. Promoter and piggyBac activities within embryos of the potato tuber moth, Phthorimaea operculella, Zeller (Lepidoptera: Gelechiidae). *Gene* **342**: 293-301.

Urnov, F.D., Miller, J.C., Lee, Y.L., Beausejour, C.M., Rock, J.M., Augustus, S., Jamieson, A.C., Porteus, M.H., Gregory, P.D., Holmes, M.C., 2005. Highly efficient endogenous human gene correction using designed zinc-finger nucleases. *Nature* **435**: 646-651.
